# Hydrophobic ion pairing and microfluidic nanoprecipitation enable efficient nanoformulation of a small molecule indolamine 2, 3‐dioxygenase inhibitor immunotherapeutic

**DOI:** 10.1002/btm2.10599

**Published:** 2023-10-28

**Authors:** Parisa Badiee, Michelle F. Maritz, Pouya Dehghankelishadi, Nicole Dmochowska, Benjamin Thierry

**Affiliations:** ^1^ Future Industries Institute and ARC Centre of Excellence Convergent Bio‐Nano Science and Technology University of South Australia Adelaide Australia; ^2^ UniSA Clinical and Health Sciences University of South Australia Adelaide Australia

**Keywords:** cancer immunotherapy, hydrophobic ion pairing, immune checkpoint inhibitors, indoleamine 2, 3‐dioxygenase, nanomedicine

## Abstract

Blockade of programmed cell death‐1 (PD‐1) is a transformative immunotherapy. However, only a fraction of patients benefit, and there is a critical need for broad‐spectrum checkpoint inhibition approaches that both enhance the recruitment of cytotoxic immune cells in cold tumors and target resistance pathways. Indoleamine 2, 3‐dioxygenase (IDO) small molecule inhibitors are promising but suboptimal tumor bioavailability and dose‐limiting toxicity have limited therapeutic benefits in clinical trials. This study reports on a nanoformulation of the IDO inhibitor navoximod within polymeric nanoparticles prepared using a high‐throughput microfluidic mixing device. Hydrophobic ion pairing addresses the challenging physicochemical properties of navoximod, yielding remarkably high loading (>10%). The nanoformulation efficiently inhibits IDO and, in synergy with PD‐1 antibodies improves the anti‐cancer cytotoxicity of T‐cells, in vitro and in vivo. This study provides new insight into the IDO and PD‐1 inhibitors synergy and validates hydrophobic ion pairing as a simple and clinically scalable formulation approach.

**Translational Impact Statement**

The therapeutic indexes of small molecule immunotherapy drugs are often limited by their physicochemical properties, and novel formulation approaches are required to harness their full potential. Here we demonstrate that hydrophobic ion pairing and microfluidic nanoformulation is an efficient approach to address the challenging physicochemical properties of the indoleamine 2, 3‐dioxygenase (IDO) inhibitor navoximod. The IDO nanoformulations showed potent anti‐tumor activity and synergize with PD‐1/PD‐L1 checkpoint inhibition. This novel and translatable approach has the potential to enable the design of much needed broad‐spectrum checkpoint inhibition immunotherapy.

## 1 INTRODUCTION

The emergence of immune checkpoints inhibitors such as antibodies against programmed cell death‐1 (PD‐1) has dramatically transformed the landscape of cancer therapies.^1^ PD‐1 receptors are upregulated upon activation of T‐cells and interactions with their ligands (such as PD‐L1) lead to T‐cell exhaustion.^2^ This pathway of T‐cell exhaustion is mediated through the overexpression of PD‐L1 in many tumor cells and immunosuppressive immune cells.^3^ Treatment with antibodies against PD‐1 (aPD‐1) has been shown to restore the cytotoxic activity of the immune system by blocking the PD‐1/PD‐L1 pathway, enhancing the cytotoxicity and proliferation of T‐cells.^4^, ^5^

**FIGURE 1:**
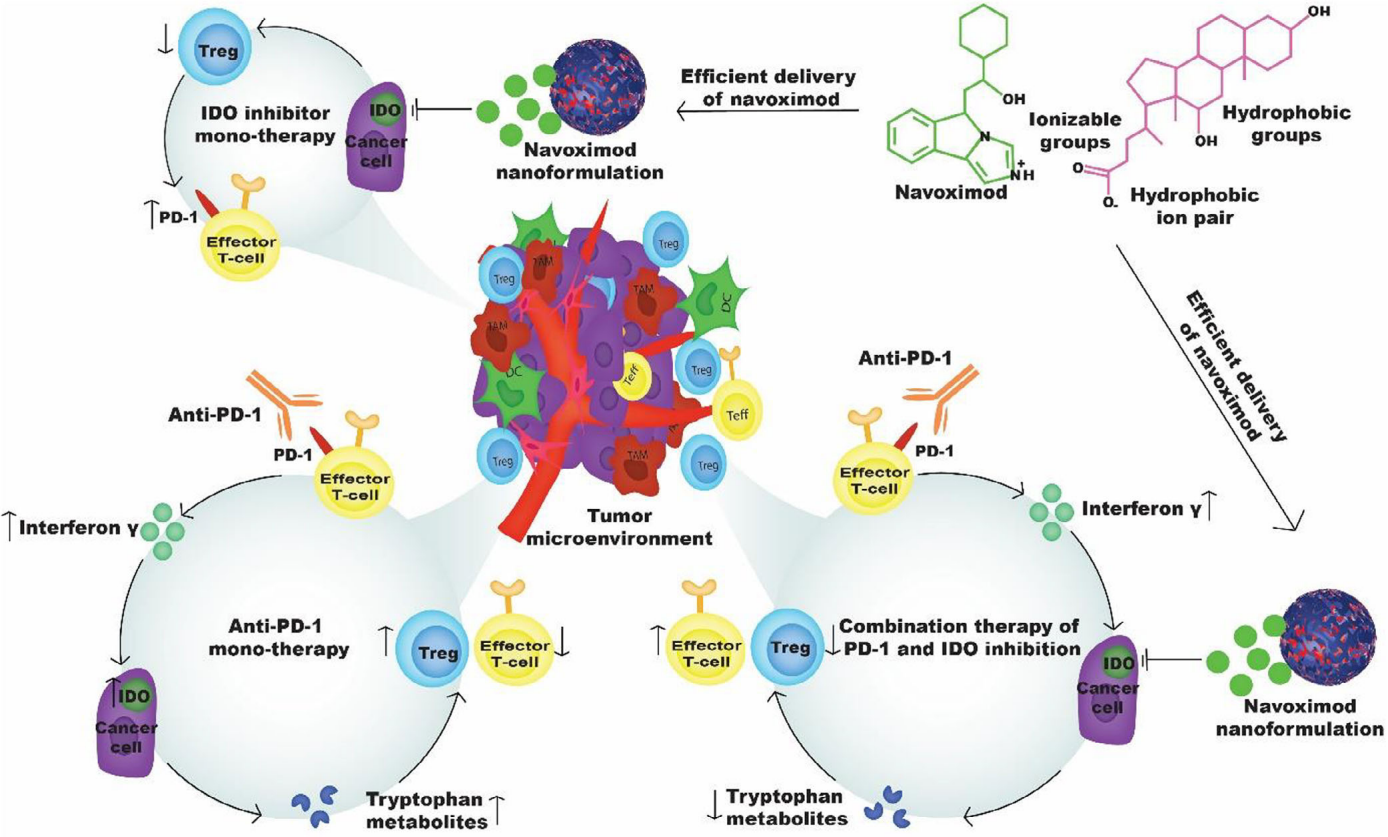
Schematic illustration of mechanisms underpinning highly potent anti‐tumor effects for combined indoleamine 2, 3‐dioxygenase (IDO) and PD‐1 inhibition. Hydrophobic ion pairing enables efficient nanoformulation of the IDO inhibitor navoximod. However, monotherapy using navoximod nanoformulation results in overexpression of PD‐1 receptors and moderate anti‐tumor response in vivo. Conversely, monotherapy with PD‐1 antibodies (anti‐PD‐1) results in overexpression of the IDO enzyme which is thought to cause resistance to the therapy. This can be overcome by combination therapy with the navoximod nanoformulation and anti‐PD‐1 checkpoint inhibition.

The patient response rate to PD‐1/PD‐L1 antibodies is, however, modest and the majority of patients do not benefit. This is mainly due to both insufficient tumor immunogenicity and the development of resistance.^6^ Although the mechanisms of resistance to PD‐1/PD‐L1 inhibition are yet to be fully elucidated, it is likely that the overexpression of other immune checkpoints is a contributing factor.^7^ In this regard, inhibitors of indoleamine 2, 3‐dioxygenase (IDO) are under clinical investigation, particularly in combination with PD‐1/PD‐L1 inhibitors because of the observed correlation between the expression of PD‐1 and IDO immune checkpoints in patient‐derived tumor tissues and the possible role of IDO in inducing resistance to inhibitors of the PD‐1/PD‐L1 pathway.^8^, ^9^ IDO is an enzyme that catabolizes tryptophan to kynurenine and other metabolites.^10^ The decrease of tryptophan, and the subsequent increase of its metabolites, decrease the proliferation and activity of immune cells, leading to immune cell suppression.^10^ Upregulation of IDO in cancer cells and some immune cells is mediated by inflammatory cytokines such as interferon (INF)‐γ, and aPD‐1 therapy can therefore increase IDO expression due to the increased release of INF‐γ by restored cytotoxic T‐cells, which counteracts the therapeutic benefit of aPD‐1 therapy.^11^ There is therefore a strong mechanistic rationale for combining immune checkpoint inhibition of the PD‐1 pathway with inhibition of IDO (Figure 1), where overexpression of IDO caused by blockade of the PD‐1/PD‐L1 pathway and secretion of INF‐γ is efficiently inhibited. The resulting high level of tryptophan may reduce the number of regulatory T‐cells (Treg) and promote the proliferation of T‐cells, leading to higher anti‐tumor immunity.

Navoximod (NLG‐919, GDC‐0919) is a potent IDO small molecule inhibitor which has been evaluated both as a single therapy and in combination with other cancer therapies in preclinical and clinical studies.^12^, ^13^ As a small molecule, navoximod has a short half‐life and high off‐target biodistribution, which is usually associated with resistance and side effects.^14^ Furthermore, due to its low oral bioavailability, navoximod is typically administered at high doses, resulting in high rates of adverse effects.^15^ Finally, navoximod either as a single or dual therapy with PD‐L1 antibodies yielded only modest efficacy in clinical trials, despite the high potency of navoximod in priming anti‐tumor immune activity, highlighting the insufficient tumor bioavailability of this potent molecule in the current administration paradigm.^13^, ^16^ Complete or partial response was not observed in any of the patients in one of the clinical trials and was only observed in 11% of patients in the other trial. Toward gaining insights into the therapeutic potential of IDO inhibitors such as navoximod alone or in combination with checkpoint inhibition and, if warranted, supporting the development of new broad spectrum checkpoint inhibition algorithms, pharmaceutical formulations that improve their tumor bioavailabilities are needed.

Toward addressing this need, several IDO inhibitor nanoformulations have been recently proposed, yielding encouraging preclinical efficacy data.^17^, ^18^, ^19^ A common feature of these nanoformulations is their reliance on chemical alteration or derivatization of the small molecule IDO inhibitors, which is necessary to overcome their challenging physicochemical properties and enhance the formulation of these drugs within polymeric nanoparticles (NPs).^20^, ^21^, ^22^ Loading of weakly hydrophobic (LogP <3.5) small molecules within nanoparticles is indeed challenging due to insufficient supersaturation during the mixing of solvent and anti‐solvent, which is necessary in nanoprecipitation routes.^23^ Despite their merits, such approaches add substantial complexity to the formulation. In addition, modification of the parent inhibitor can also negatively affect its activity.^24^ An alternative promising strategy to improve the drug loading and stability of drugs with challenging physicochemical properties is hydrophobic ion pairing.^25^ In hydrophobic ion pairing, a drug that is not intrinsically hydrophobic enough to be efficiently loaded within polymeric NPs is complexed with a counter ion molecule to increase its apparent hydrophobicity, consequently improving loading within polymeric NPs. Unlike the synthesis of prodrugs, this approach does not permanently modify the drug chemically and therefore preserves its biological activity.

This study aimed to better understand the benefits of combined IDO inhibition and checkpoint inhibition using a simple yet efficient nanoformulation of navoximod within Poly(ethylene glycol)‐b‐poly(lactide‐co‐glycolide) (PEG‐PLGA) NPs using hydrophobic ion pairing. PEG‐PLGA was selected owing to its well‐established safety profile and extensive use for the preparation of nanoformulations including clinically.^26^, ^27^ In addition, microfluidic‐based nanoprecipitation was used as it enables simpler and more reproducible manufacturing of PEG‐PLGA nanocarriers compared to conventional bulk mixing methods.^28^ We validated the efficacy of the nanoformulation in inhibiting IDO in head and neck cancer (HNC) cells and subsequently demonstrated the synergistic efficacy of the navoximod nanoformulation in combination with aPD‐1 to overcome resistance to aPD‐1 in vitro and in vivo using a murine orthotopic HNC model.

## 2 MATERIALS AND METHODS

### 2.1 Materials

PLGA (15–25 KDa; lactic acid/glycolic acid: 50/50) and methoxy‐PEG‐PLGA (PEG‐PLGA) (2–15 KDa; lactic acid/glycolic acid: 50/50) were obtained from PoliSciTech (USA). Dimethyl sulfoxide (DMSO), acetonitrile and sodium deoxycholate were purchased from Sigma Aldrich (USA). Navoximod was from MedChemExpress (USA) and Adooq Bioscience (USA). Recombinant human INF‐γ and interleukin‐2 (IL‐2) were purchased from Peprotech (USA). Anti‐human PD‐1 antibody (clone EH12.2H7) and anti‐mouse CD279 (PD‐1) antibody (clone 29F.1A12) were sourced from Biolegend (USA). Anti‐human CD28 antibody (clone CD28.2) and anti‐human CD3 (clone OK3) were purchased from ThermoFisher (USA).

### 2.2 Preparation of navoximod nanoformulation

**FIGURE 2:**
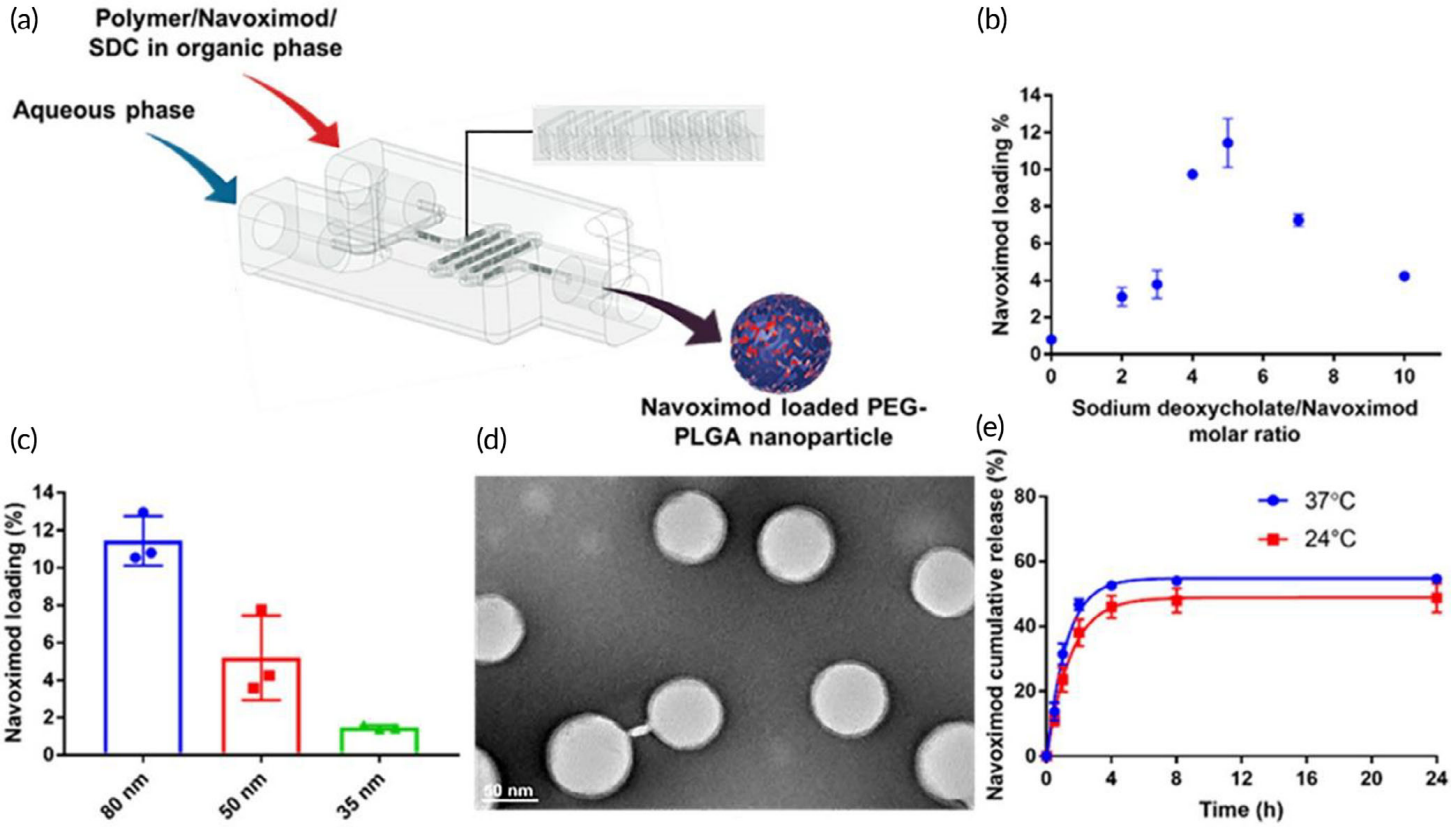
Optimization and physicochemical characterization of the navoximod nanoformulation. (a) Schematic diagram of navoximod nanoformulation preparation using a 3D printed microfluidic device for nanoprecipitation; SDC, Sodium deoxycholate. Effect of (b) sodium deoxycholate/navoximod molar ratio and (c) NPs' size on drug loading. (d) Representative TEM image of the navoximod nanoformulation (scale bar is 50 nm). (e) Cumulative drug release profile of navoximod nanoformulation over 24 h at 24 and 37°C.

PEG‐PLGA NPs were synthesized utilizing a 3D printed microfluidic chip (Figure 2a) which was fabricated using a MiiCraft 3D printer (Rays Optics, Canada) with the Master Mold resin (Creative CADworks, Canada). The mixing channel consists of 13 mixing units that form a herringbone structured channel with dimensions of 0.6 and 1.1 mm for width and height, respectively. Aqueous and polymer phases were introduced through two inlets and mixed in the herringbone structured mixing channel to nano‐precipitate the polymer. To synthesize the navoximod nanoformulation, a sodium deoxycholate solution in DMSO and navoximod in ethanol were premixed at different molar ratios as indicated in Table 1. To optimize the navoximod loading, the navoximod/sodium deoxycholate mixture was added to the polymer mixture (10 mg/mL) in DMSO with various navoximod/polymer weight ratios (Table 1). The polymer mixture was comprised of 50% PLGA and 50% PEG‐PLGA, and Milli‐Q® water was used as the aqueous phase. The flow rates of water and DMSO were controlled by two syringe pumps (Chemyx fusion 200). A range of flow rates (aqueous/solvent: 3/1, 8/1, 15/3 mL/min) was tested to optimize the physicochemical properties of the NPs. The navoximod nanoformulation was collected from the outlet of the device and centrifuged and washed at 24000*g* for 15 min at 4°C and redispersed in Milli‐Q® water for further analysis. For the small sized NPs, the sample was washed and collected using centrifugal filters with a 10 KDa cut‐off (Amicon® Ultra‐4, Merck Millipore, USA) instead of centrifugation. Navoximod loaded nanoparticles without hydrophobic ion pairing were prepared as per the above procedure without adding sodium deoxycholate into the polymer mixture.

**TABLE 1:** Optimization of navoximod nanoformulation by hydrophobic ion pairing and nanoprecipitation.

Navoximod/polymer ratio (W/W %)	Sodium deoxycholate/navoximod (mol/mol)	Aqueous/Solvent flow rate (mL/min)	Drug loading (W/W %, mean ± SD)	Hydrodynamic diameter (nm, mean ± SD)	Polydispersity index (PDI ± SD)
20	0	3/1	0.8 ± 0.1	43.5 ± 8.7	0.12 ± 0.01
10	3/1	3/1	1 ± 0.3	52.5 ± 1.4	0.10 ± 0.02
10	5/1	3/1	0.8 ± 0.1	66 ± 7.8	0.10 ± 0.01
10	10/1	3/1	1.7 ± 0.9	76.8 ± 6.4	0.09 ± 0.03
20	2/1	3/1	5.8 ± 3.1	63.5 ± 2.1	0.13 ± 0.03
20	3/1	3/1	5.8 ± 2.1	78 ± 0.7	0.15 ± 0.08
20	4/1	3/1	9.7 ± 0.2	83.5 ± 2.5	0.10 ± 0.00
20	5/1	3/1	10.1 ± 0.4	80.8 ± 5.0	0.12 ± 0.02
20	7/1	3/1	7.2 ± 0.3	96 ± 2.8	0.11 ± 0.02
20	10/1	3/1	6.0 ± 3.1	109 ± 12.7	0.19 ± 0.06
20	5/1	8/1	5.2 ± 2.2	51.4 ± 1.3	0.12 ± 0.02
20	5/1	15/3	1.5 ± 0.1	36.9 ± 0.7	0.16 ± 0.01

### 2.3 Physicochemical and biological characterization of navoximod nanoformulation

The details of the methodology used to characterize the navoximod nanoformulation and investigate the in vitro and in vivo biological effects of navoximod nanoformulation/aPD‐1 are described in the supporting information. All animal work was approved by the University of South Australia Animal Ethics Committee (approval U20‐21) and was performed in accordance with the Animal Welfare Act and the Australian Code for the Care and Use of Animals for Scientific Purposes.

### 2.4 Statistics

Experiments were independently repeated at least twice. Data were analyzed using a one‐way ANOVA with Tukey post hoc test using GraphPad Prism 7 (GraphPad Software, Inc., USA) unless stated otherwise. For the animal study, tumor bearing mice were divided into the treatment groups based on the tumor volume so that the average tumor volume was approximately 50 mm^3^ per group. The number of animals (*N* = 5 or 6 per group) was selected based on the previously published studies to be properly powered for an efficacy study. Statistical significance was indicated by asterisks as *p < 0.05; **p < 0.01; ***p < 0.001; ****p < 0.0001 and ns indicates not significant. The data are expressed as average ± standard error of mean.

## 3 RESULTS AND DISCUSSION

### 3.1 Hydrophobic ion pairing improved the physicochemical characteristics of navoximod nanoformulation

The loading of molecules with challenging physicochemical characteristics within PLGA and PEG‐PLGA NPs is suboptimal,^29^ and as expected our initial attempts at loading navoximod within PEG‐PLGA NPs yielded very low efficiency (Table 1). We therefore employed hydrophobic ion pairing to increase the compatibility between the drug and the hydrophobic core of the PEG‐PLGA NPs (Figure 2a; Figure S1). The complexation of the small molecule navoximod with a more hydrophobic counter ion is used to increase the hydrophobicity of the small molecule which in turn increases the possibility of achieving sufficient supersaturation by the mixing of solvent and anti‐solvent during the nanoprecipitation. Sodium deoxycholate was utilized as the counter ion. To optimize the drug loading and physicochemical properties of the NPs, key formulation parameters namely the ratios of navoximod/polymer (w/w), sodium deoxycholate/navoximod (mol/mol) and flow rates of aqueous/solvent phases were optimized. The details of each tested condition and their effects on drug loading, the NPs' number‐based hydrodynamic size and polydispersity index are summarized below (Table 1). The graphs of intensity‐based size distribution of the tested nanoformulations are shown in Figure S2.

By increasing the weight ratio of navoximod/polymer without changing any other parameters, the drug loading increased, with a 20% ratio yielding the highest loading. As shown in Figure 2b, at 20% navoximod/polymer, the loading was further enhanced by increasing the sodium deoxycholate/navoximod ratio up to 5/1. Further increase in the sodium deoxycholate/navoximod ratio was associated with a decrease in the loading of navoximod. To optimize the NPs' size, various aqueous/solvent flow rates were tested. The microfluidic nanoprecipitation device used in this study made it possible to easily adjust the NPs size by adjusting the flow rates without changing the polymer composition or concentration. Keeping constant both the navoximod/polymer (20%) and sodium deoxycholate/navoximod (5/1) ratios, very small PEG‐PLGA NPs could be reliably prepared at a high flow rate, but this was associated with a significant decrease of the drug loading (Figure 2c). The highest drug loading was achieved at ratios of 20% navoximod/polymer, 5/1 sodium deoxycholate/navoximod, and 3/1 aqueous/solvent flow rate. The drug loading and NPs' size at these optimized parameters were 10.1 ± 0.4% and 80.8 ± 5 nm, respectively. Nanoformulations within this hydrodynamic diameter range have been commonly used in preclinical cancer immunotherapy investigations,^30^ and these conditions were therefore selected and used for subsequent studies. The data demonstrate the remarkable efficiency of hydrophobic ion pairing as it improved the loading of navoximod from less than 1% to above 10%. This is a significant improvement in the loading of navoximod within PEG‐PLGA nanoparticles, which is achieved without doing any chemical modification in the backbones of the polymer or drug. Considering the well reported poor loading within PEG‐PLGA nanoparticles of small drugs with limited hydrophobicity, typically only a few percent in the literature,^31^ the achieved 10% drug loading is excellent. The optimized navoximod nanoformulation was highly monodispersed with a narrow hydrodynamic size distribution, and was stable over 3 days (Figure S3). The zeta potential of the navoximod nanoformulation was −23.1 ± 0.5 mV (Figure S4), which might be due to the carboxylate end cap of the polymer. TEM confirmed the monodispersity and spherical morphology of the nanoformulation (Figure 2d) as well as the size (71.9 ± 1.6 nm obtained by evaluating the size of 60 NPs in 10 TEM images). We had previously confirmed the stability of PEG‐PLGA nanoparticles prepared with a similar polymeric composition in 10% and 50% serum.^32^ The PEG‐PLGA nanoparticles were stable at both 25 and 37°C over 1 week of study in 10% and 50% fetal bovine serum without any significant change in the size and PDI of nanoparticles.

Next, the release of the navoximod from the nanoformulation was determined under sink condition and at temperatures of 37 and 24°C. As expected, the navoximod nanoformulation displayed a biphasic release profile at both temperatures, where a high‐rate release was observed over the first 2 h of the study followed by a slower release phase (Figure 2e). At the 2 h time‐point 48.4% (at 37°C) and 33.8% (at 24°C) of the drug was released, which can be accredited to the drugs adsorbed on the surface of NPs or were close to the surface.^33^ Subsequent time points demonstrated a sustained drug release, where 54.6% (at 37°C) and 48.8% (at 24°C) of drug was released over 24 h. The navoximod nanoformulation demonstrated faster release rate at 37°C compared to 24°C. This can be due to higher diffusion rate of the drug at higher temperatures, which is the main mechanism of drug release in the initial release phase.^33^

Altogether, the data validated the use of hydrophobic ion pairing and microfluidic nanoprecipitation toward the preparation of high quality navoximod nanoformulations. Hydrophobic ion pairing is actively being applied for the nanoformulation of small molecule drugs and has been shown to improve drug loading, stability, and release profiles.^24^, ^34^ In our hand, hydrophobic ion pairing of navoximod with sodium deoxycholate as the pairing agent dramatically improved its loading within the PEG‐PLGA NPs. A variety of approaches have been successfully explored for the formulation of IDO inhibitors, including drug dimerization and conjugation of the drug to peptide, polymer, or lipid.^17^, ^18^, ^19^, ^21^, ^35^, ^36^ For example, formulation of navoximod using self‐assembling peptides targeting PD‐L1 increased the anti‐tumor activity of immune cells and reduced tumor size in a murine model of melanoma.^15^ Chen et al. prepared a prodrug of navoximod using PEG and self‐assembling micelles.^21^ A similar method was used by Liu et al. who PEGylated the Epacadostat IDO inhibitor with a peptide linker and co‐delivered Epacadostat and a photosensitizer using NPs.^20^ In another study, a homodimer of navoximod was prepared through the addition of a disulfide bond, which was subsequently incorporated into PEGylated oxaliplatin for sequential release in the acidic tumor microenvironment.^19^ Lipid tagged or gelatin conjugated with the indoximod IDO inhibitor were also employed to enable loading within liposomes and mesoporous silica NPs, respectively.^18^, ^35^, ^37^ Despite the merits of these approaches, chemical modification of IDO inhibitors introduces additional manufacturing and regulatory barriers to translation. Combining hydrophobic ion pairing and nanoprecipitation using microfluidic devices is therefore promising toward the manufacturing of high quality nanoformulations, especially considering the recent development of GMP compatible microfluidic nanoprecipitation technology.

### 3.2 Navoximod nanoformulation effectively inhibits IDO activity

**FIGURE 3:**
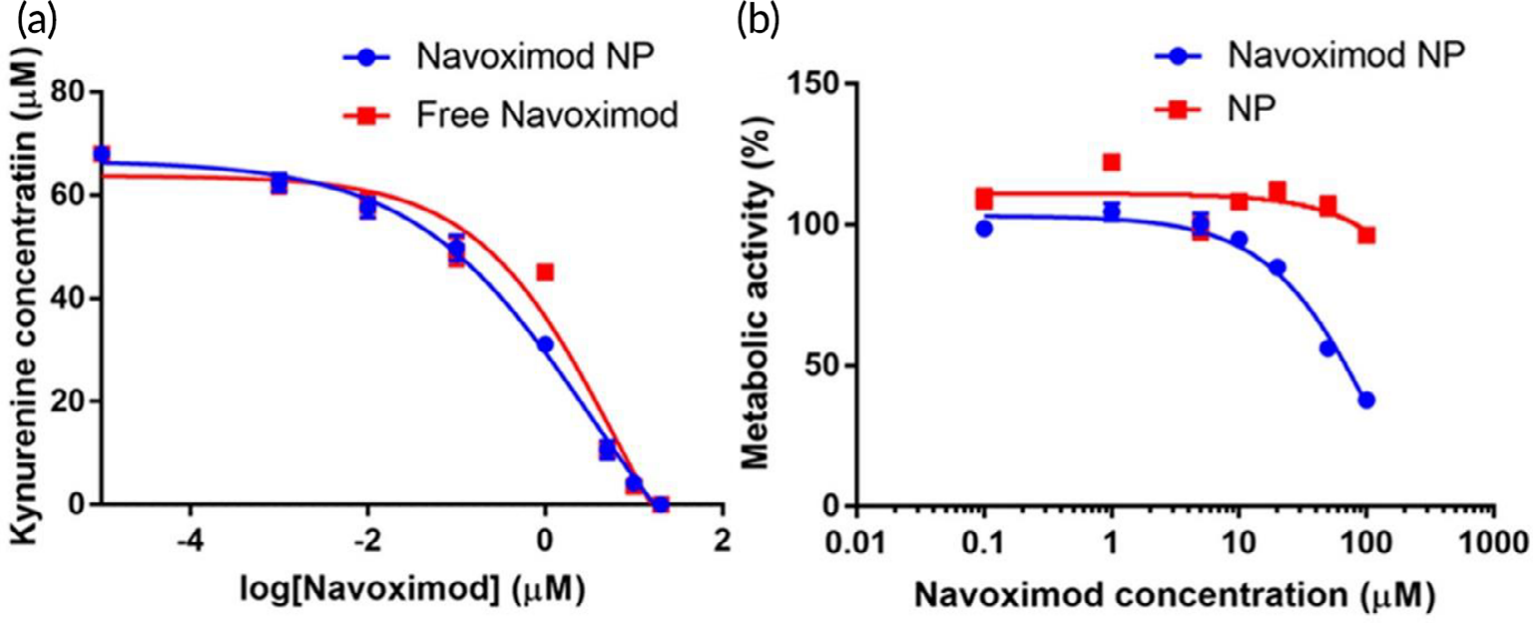
Inhibition of IDO enzyme and effect on UM‐SCC1 cells metabolic activity. (a) The effect of navoximod nanoformulation (Navoximod NP) and free navoximod on the inhibition of IDO enzyme in UM‐SCC1 cells (*N* = 5/navoximod concentration). (b) The effect of Navoximod NPs and blank NPs on the metabolic activity of UM‐SCC1 cells by MTT assay (*N* = 5/navoximod concentration).

To confirm the efficiency of the navoximod nanoformulation to inhibit the IDO enzyme, an IDO activity assay was performed in the UM‐SCC1 cell line used as a model of human head and neck cancer squamous cell carcinoma (HNSCC). UM‐SCC1 cells were treated with several concentrations of navoximod nanoformulation or free navoximod (as a control) and the concentration of kynurenine produced by IDO activity was measured. The navoximod nanoformulation efficiently inhibited the activity of IDO with an IC50 of 3.33 μM compared to an IC50 of 6.66 μM for the free navoximod (Figure 3a). This assay also provided insight into the minimum dose required for in vitro biological assays. It also demonstrated that navoximod loading by hydrophobic ion pairing with sodium deoxycholate did not affect its function. This is not surprising as the counter ion interacts with the drug molecule only physically.^25^ Likewise, when navoximod was nanoformulated within cyclodextrin, the activity of the drug was not altered compared to the free drug.^22^ In contrast, chemical modification of navoximod was previously shown to be associated with a reduction of IDO inhibition.^21^ In addition, the effect of the navoximod nanoformulation on the metabolic activity of UM‐SCC1 cells was analyzed by 3‐(4,5‐dimethylthiazol‐2‐yl)‐2,5‐diphenyltetrazolium bromide (MTT) assay and compared to that of the blank NPs. No changes in the metabolic activity were observed up to 10 μM of navoximod nanoformulation (Figure 3b). Therefore, 10 μM navoximod nanoformulation was selected as it is above the observed IC50 for inhibition of the IDO activity, but did not affect the cancer cell's metabolic activity.

### 3.3 Navoximod nanoformulation/aPD‐1 significantly reduced the immunosuppressive regulatory T‐cells and enhanced the cytotoxicity of T‐cells

**FIGURE 4:**
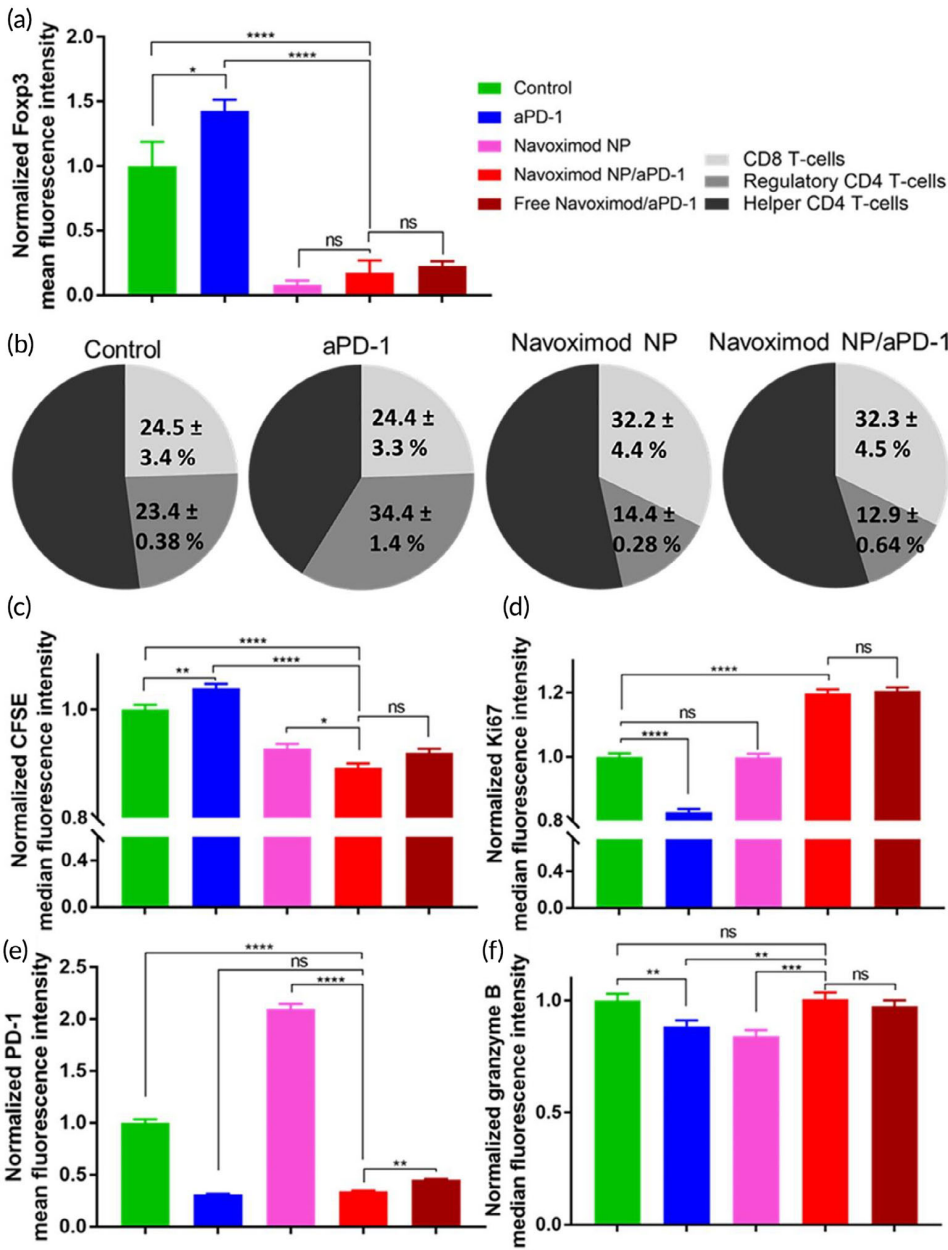
Effect of the navoximod nanoformulation as single or combination therapy with aPD‐1 on immunosuppressive and cytotoxic characteristics of T‐cells. (a) Effect of treatment of co‐cultures of activated T‐cells and UM‐SCC1 cells with aPD‐1 (5 μg/mL), navoximod nanoformulation (navoximod NP) (10 μM), navoximod NP combined with aPD‐1 (10 μM and 5 μg/mL), and free navoximod combined with aPD‐1 (10 μM and 5 μg/mL) on regulatory T‐cells (CD3^+^CD4^+^FoxP3^+^ T‐cells), analyzed 48 h after treatment. (b) Effect of treatment on the populations of cytotoxic CD8 T‐cells (CD3^+^CD8^+^ T‐cells), regulatory T‐cells (CD3^+^CD4^+^FoxP3^+^ T‐cells), and helper CD4 T‐cells (CD3^+^CD4^+^FoXP3^−^ T‐cells). (c) Proliferation of CFSE labeled T‐cells in co‐culture of activated T‐cells and UM‐SCC1 cells 48 h post treatment. (d) Ki67 expression measured 24 h after treatment. (e) Expression of PD‐1 receptors in treated T‐cells 48 h after treatment. (f) Impact of treatment on granzyme B production by T‐cells 24 h after treatment. The cellular markers were measured by imaging flow cytometry after gating for CD3^+^ single cells. Data were analyzed using a one‐way ANOVA with Tukey post hoc test.

The biological effects of the navoximod nanoformulation as either a single therapy or in combination with aPD‐1 to enhance immune checkpoint inhibition were next investigated. The main role of IDO inhibition is to reduce the immunosuppressive regulatory T‐cells (Treg) population. IDO mediated increase of Treg expression is involved in low survival both in preclinical tumor models and cancer patients.^38^, ^39^, ^40^, ^41^ Reduction in the population of Treg cells was observed in a preclinical murine model of pancreatic cancer co‐treated by PD‐L1 antibodies and self‐assembled peptide conjugated navoximod.^17^ To determine the impact of the navoximod nanoformulation, the Treg population was analyzed by imaging flow cytometry in a T‐cells/UM‐SCC1 cells co‐culture. Figure 4a shows that the blockade of PD‐1 receptors by aPD‐1 significantly increased the mean fluorescence intensity of Treg cells by 42.7%. The navoximod nanoformulation as a single treatment dramatically decreased the mean fluorescence intensity of Treg cells population by 91.9%. Similarly, treatment of the co‐culture with navoximod nanoformulation in combination with aPD‐1 reduced the Treg cells' mean fluorescence intensity by 82.8% compared to the control (no treatment). This data demonstrate that the navoximod nanoformulation efficiently overcame aPD‐1 induced increase in Treg cells. Cytotoxic CD8 T‐cells and the ratio of CD8 T‐cells to Treg cells are also critical to the anti‐tumor activity of the immune system. Treatment with the navoximod nanoformulation alone or in combination with aPD‐1 increased the number of CD8 T‐cells, which led to higher ratios of CD8 T‐cells/Treg cells relative to the no treatment control group (Figure 4b). The ratio of CD8 T‐cells to Treg cells increased to 2.2 and 2.5 upon navoximod nanoformulation and navoximod nanoformulation/aPD‐1 combination treatment, respectively, compared to the ratios of no treatment control (1) and aPD‐1 single therapy (0.7). These results show that the combination of navoximod nanoformulation and aPD‐1 treatment efficiently mitigated the aPD‐1 induced increase of Tregs and decrease of CD8/Treg cells, implicating a role for IDO upregulation. The connection between the PD‐1 and IDO pathways is reported to contribute toward resistance to aPD‐1 therapy.^42^

To investigate the impact of treatment with the navoximod nanoformulation and navoximod nanoformulation/aPD‐1 combination therapy on T‐cell proliferation, T‐cells were labeled with the proliferation marker carboxyfluorescein succinimidyl ester (CFSE). aPD‐1 treatment decreased proliferation, as observed by a modest increase in the CFSE median fluorescence intensity compared to the untreated cells (4%, Figure 4c). This might be due to the upregulation of the IDO enzyme upon aPD‐1 treatment. Consistent with our results, a previous study observed that aPD‐1 treatment increases Treg cells, which consequently reduced the expansion of cytotoxic T‐cells.^43^ On the other hand, IDO inhibition by the navoximod nanoformulation significantly decreased T‐cell CFSE fluorescence intensity (7%, compared to untreated cells), indicating increased T‐cell proliferation (Figure 4c). T‐cell proliferation was further significantly increased in the case of the dual navoximod nanoformulation/aPD‐1 therapy (11% vs. 7%, *p* < 0.05).

In line with the reduced CFSE proliferation result, Ki67 as a marker of T‐cells' proliferation was quantified by imaging flow cytometry. This confirmed that aPD‐1 treatment reduced the proliferation of T‐cells compared to the untreated cells by the decrease of Ki67 median fluorescence intensity (18%, Figure 4d). Although the navoximod nanoformulation had no significant effect on the fluorescence intensity of Ki67 in T‐cells, the navoximod nanoformulation/aPD‐1 combination therapy significantly increased the median fluorescence intensity of Ki67 in T‐cells by 19% compared to untreated T‐cells, confirming the higher T‐cell proliferation observed in the CFSE assay.

PD‐1 receptor expression was next quantified as a marker of T‐cell exhaustion. As expected, treatment with aPD‐1 effectively inhibited PD‐1 receptors and decreased the mean fluorescence intensity of PD‐1 receptors by ~69% (Figure 4e). Interestingly, navoximod nanoformulation treatment induced a two‐fold greater increase in the PD‐1 mean fluorescence intensity, demonstrating overexpression of PD‐1 receptors, due to increased T‐cells cytotoxicity upon IDO inhibition. This observed increase of PD‐1 receptors can also implicate resistance to IDO inhibition single therapy. However, the addition of aPD‐1 to navoximod nanoformulation treatment efficiently inhibited PD‐1 receptors, as comparable to aPD‐1 single therapy.

Secretion of Granzyme B, which is required for cell lysis and apoptosis is a well‐established marker of T‐cell cytotoxicity.^44^ A single treatment with aPD‐1 or navoximod nanoformulation induced lower levels of granzyme B fluorescence intensity of T‐cells, suggesting overexpression of the alternative immune checkpoints following inhibition with either single therapy (Figure 4f). The combination of navoximod nanoformulation and aPD‐1 however, successfully restored the level of granzyme B signal of T‐cells and the cytotoxicity of T‐cells (Figure 4f).

It is noteworthy that in all these experiments, the efficacy of navoximod nanoformulation was analogous to that of the free navoximod, confirming that the nanoformulation of navoximod using hydrophobic ionic pairing had no effect on the stability and activity of the drug. In addition, the PEG‐PLGA NPs did not show an effect on the metabolic activity of T‐cells, as the luminescence of metabolically active T‐cells following treatment with drug‐free NPs was comparable with that of the control group according to the RealTime‐Glo™ MT cell viability assay (Figure S5). This confirms that the observed biological effects are solely related to the pharmacologic activity of navoximod, not the carrier.

### 3.4 Improved in vitro anti‐tumor effects by navoximod nanoformulation/aPD‐1 combination therapy

**FIGURE 5:**
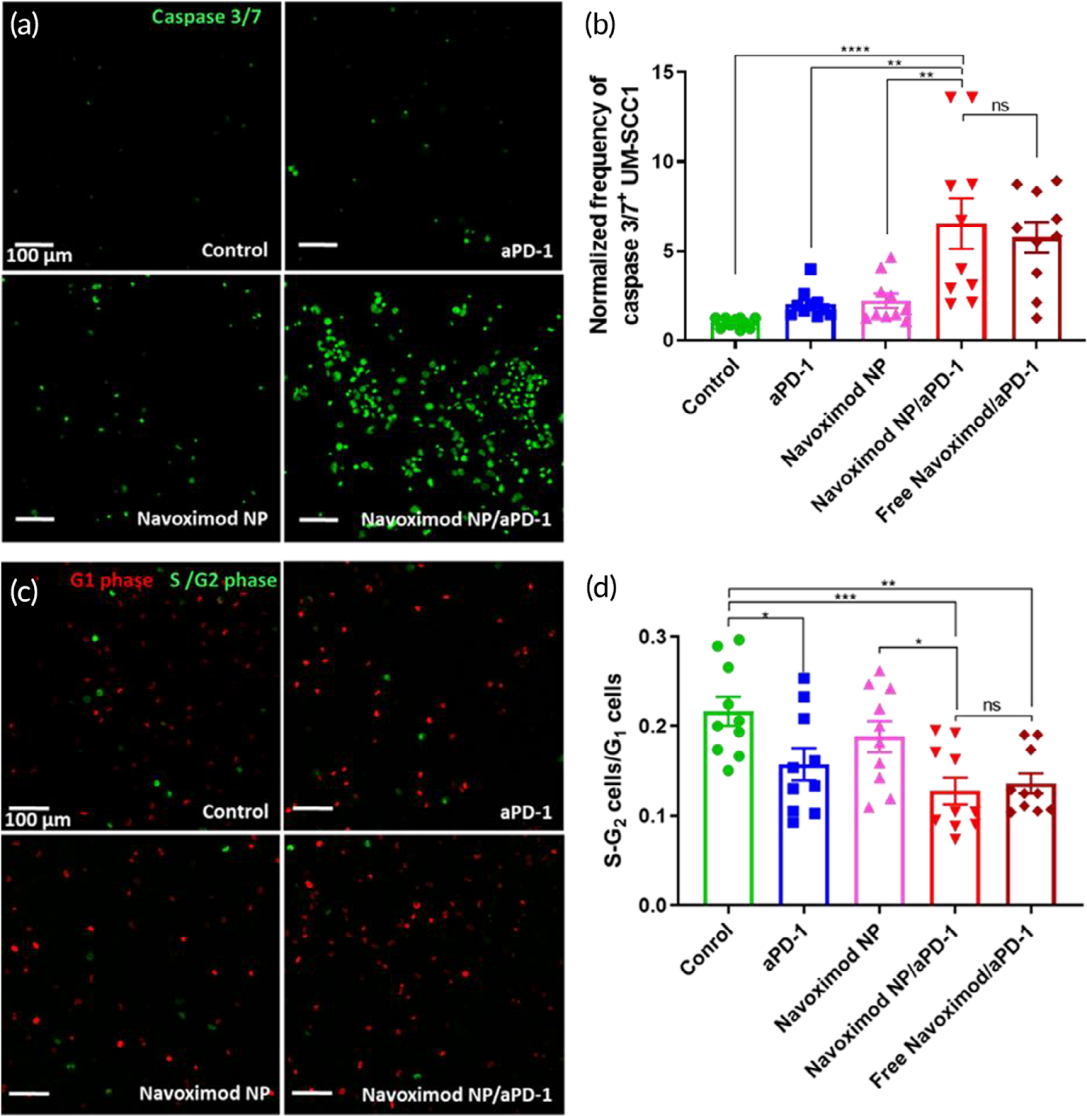
Effect of the navoximod nanoformulation as a single or combination therapy with aPD‐1 on the activity of T‐cells against UM‐SCC1 cells. (a) Representative images and (b) quantification of caspase 3/7 activated UM‐SCC1 cells co‐cultured for 24 h with T‐cells after treatment with aPD‐1 (5 μg/mL), navoximod nanoformulation (Navoximod NP, 10 μM), Navoximod NPs combined with aPD‐1 (10 μM and 5 μg/mL), and free navoximod combined with aPD‐1 (10 μM and 5 μg/mL). (c) Representative images and (d) quantification of fucci‐SCC1 cell cycle after treatment. Red cells are in the G1 phase, green cells are in the S/G2 phase. In all images, the scale bar is 100 μm. Cell populations were quantified by counting 10 images/group taken from different areas of well plate.

To determine the anti‐cancer activity of T‐cells treated with the navoximod nanoformulation, the apoptosis of the UM‐SCC1 cells in the co‐culture was investigated by staining for caspase 3/7 (Figure 5a). Treatment of cells by aPD‐1 or navoximod nanoformulation as a single therapy increased the population of apoptotic cancer cells by 2 and 2.2‐folds respectively, indicating the efficacy of both single treatments to increase the anti‐tumor effects of T‐cells (Figure 5b). Remarkably, a synergistic effect was observed in the navoximod nanoformulation/aPD‐1 combination therapy, which led to a 6.5‐fold increase in the number of apoptotic UM‐SCC1 cells. Combined treatment with free navoximod and aPD‐1 also increased the number of apoptotic UM‐SCC1 cells by 5.7‐fold (Figure 5b).

We finally examined the effect of the navoximod nanoformulation on the cell cycle of the UM‐SCC1 cells in co‐culture with T‐cells. The shift of cancer cells from the S‐G2 to the G1 phase of the cell cycle indicates the efficiency of T‐cells therapy in reducing cancer cell proliferation.^45^ The cell cycle response of the cancer cells was evaluated by imaging UM‐SCC1 cells that were modified to stably express fluorescent ubiquitination‐based cell cycle indicator (fucci) system (fucci‐SCC1) after treatments (Figure 5c). Representative images of fucci‐SCC1 cells 24 h after treatment are shown, where red cells represent cells in the G1 phase and green cells represent the S‐G2 phase of the cell cycle. Upon aPD‐1 treatment, the ratio of cells in S‐G2/G1 reduced to 0.157 ± 0.018 compared to 0.217 ± 0.016 in the no treatment control group, indicating G1 cell cycle arrest (Figure 5d). The navoximod nanoformulation had no significant effect on the cell cycle. On the other hand, navoximod nanoformulation/aPD‐1 combination therapy significantly decreased the ratio of S‐G2 cells/G1 cells to 0.128 ± 0.015 comparable to what was measured for treatment with free navoximod and aPD‐1 (0.136 ± 0.011). Although the effect of T‐cell on cancer cell cycle arrest was improved by combined navoximod nanoformulation/aPD‐1 treatment compared to aPD‐1 single therapy, the difference was not statistically significant.

### 3.5 Navoximod nanoformulation/aPD‐1 combination therapy efficiently improved anti‐tumor responses in an orthotopic head and neck tumor model

**FIGURE 6:**
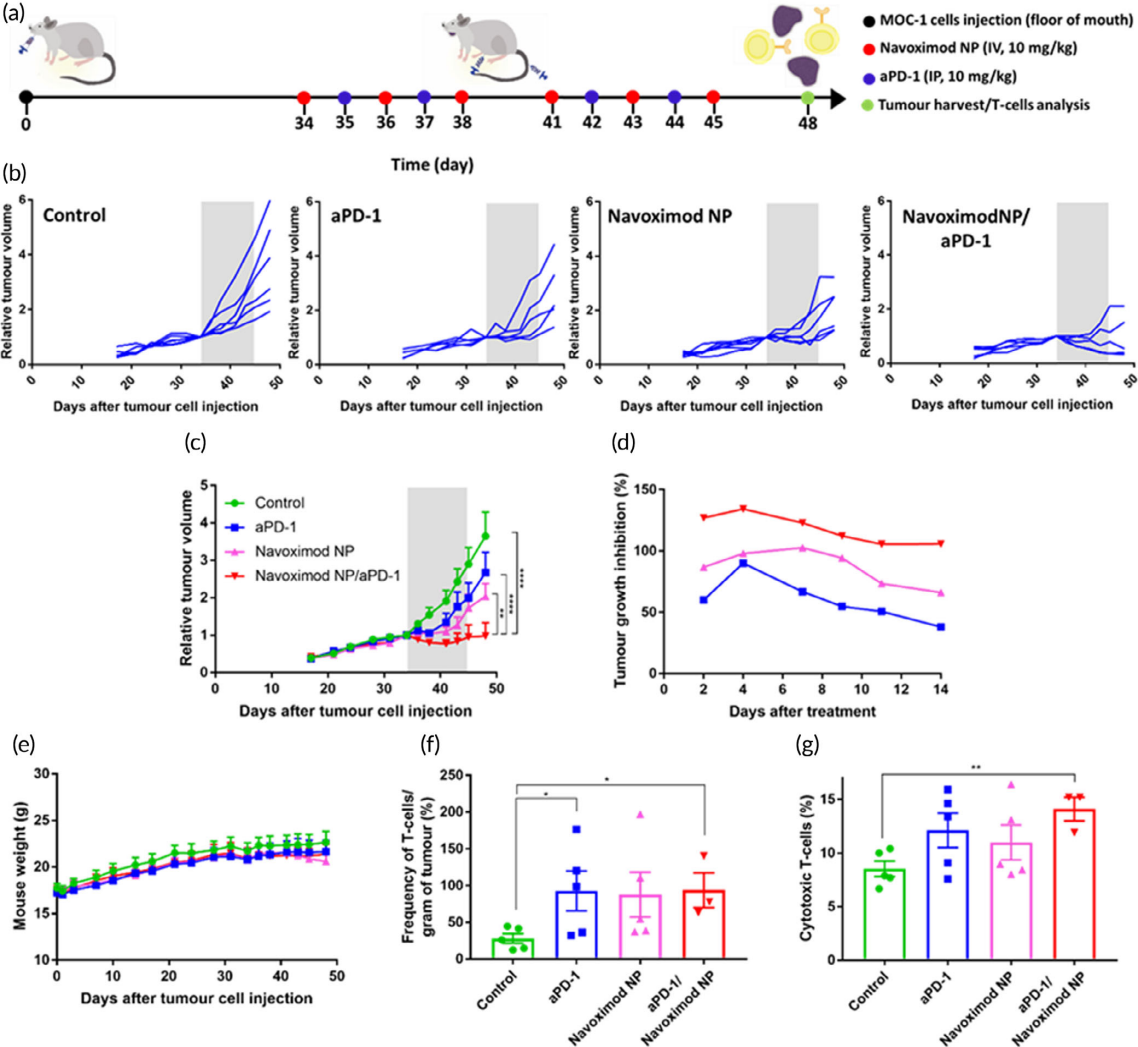
In vivo anti‐tumor effects of navoximod nanoformulation as a single or combination therapy with aPD‐1. (a) Experimental design of the animal study in orthotopic MOC‐1 bearing C57BL/6 mice. (b) Tumor volumes relative to those at the start of treatment for control (*N* = 6), aPD‐1 (*N* = 5), navoximod nanoformulation (Navoximod NP, *N* = 6), navoximod nanoformulation/aPD‐1 combination therapy (*N* = 5) groups and (C) the average tumor volumes in each group. Gray zone indicates the treatment period. Data were analyzed using a two‐way ANOVA with Tukey post hoc test. (d) Impact of treatment on tumor growth inhibition rate. (e) Tumor bearing mice body weight during the study. Effect of treatment on (f) the frequency of tumor infiltrating T‐cells per mass of tumor tissues and (g) the population of cytotoxic T‐cells in tumor tissues. T‐cells and cytotoxic T‐cells were measured by flow cytometry after gating for CD3^+^ live cells and CD8^+^ CD3^+^ live cells, respectively (*N* = 5 for control, aPD‐1 and navoximod nanoformulation, and *N* = 3 for combination therapy group due to insufficient cell number in some animals in this group). Data were analyzed by two‐tailed unpaired *t*‐test to compare the treatment with the control.

The observed increases in T‐cell proliferation, tumor cell apoptosis and cell‐cycle arrest indicated the efficiency of the combined navoximod nanoformulation/aPD‐1 therapy in enhancing T‐cell cytotoxicity against cancer cells, and encouraged us to investigate the efficacy of the navoximod nanoformulation and aPD‐1 dual therapy in vivo. To this end, an orthotopic mouse model of HNSCC was used to investigate the therapeutic effects of the navoximod nanoformulation and its synergy with aPD‐1. In this study, six doses of navoximod nanoformulation (10 mg/kg) and four doses of aPD‐1 (10 mg/kg) were administered following the establishment of the tumor in the animals' floor of mouth (Figure 6a). Single therapy of either the navoximod nanoformulation or aPD‐1 reduced the growth rate of tumors, in comparison with the control group. Remarkably, the combination therapy either stopped tumor growth or substantially reduced the growth rate (Figure 6b,c). The average tumor volume in the combination therapy group reached 52.7 ± 25.8 mm^3^ at the end of the study, compared to 97.2 ± 21.3, 114.4 ± 25.5, and 160.1 ± 37 mm^3^ for the navoximod nanoformulation, aPD‐1 and control groups, respectively. Comparing the tumor size at the end of the study to that at the start of treatment, the relative tumor volume was 0.97 in the combination therapy group, whereas it was 2, 2.7, and 3.6 for the navoximod nanoformulation, aPD‐1 and control groups, respectively. Tumor growth inhibitions were 38.1%, 66.2%, and 105.8% for single therapy aPD‐1 and navoximod nanoformulation, and combination therapy, respectively (Figure 6d). Figure S6 shows the endpoint images of tumors located at the floor of the mouth of animals which confirmed the potency of the combination therapy in inhibiting tumor growth. The calculated combination index of less than 1 (0.57) confirmed the synergistic anti‐tumor effect of the navoximod nanoformulation and aPD‐1 dual therapy. It is noteworthy that no adverse effects were observed during the study in regard to the animals' vital signs and weight (Figure 6e). There was no noticeable pathological sign in the hematoxylin and eosin staining of the heart, spleen, lung, and kidney (Figure S7). However, mild to moderate levels of anisokaryosis and very occasional binucleation was detected in liver sections of mice treated with the combination of navoximod nanoformulation and aPD‐1 (Figure S7). This is a common and non‐specific change that may indicate a low level of toxicity. Further investigation including a specific toxicology study is warranted to fully investigate the cause and extent of the observed histological changes, which can be also complemented with dose optimization studies. Further studies aimed at specifically assessing off‐target effects including immune related adverse events are warranted. In recognition of the importance of immune related adverse toxicities and the limitation of existing animal models, numerous preclinical models have been recently published.^46^, ^47^

Finally, to investigate the impact of the single and combined treatments on tumor infiltrating T‐cells, tumor tissues were collected, dissociated and stained for CD3 and CD8 surface markers. Flow cytometry analysis showed that all single and combination therapies resulted in significant increases in T‐cells numbers per tumor mass compared to the control group (Figure 6f). However, there was not a statistically significant difference between the combination therapy and single navoximod nanoformulation or aPD‐1 therapy. Further analyses of harvested T‐cells showed increases in cytotoxic CD8 T‐cells' population following all treatments, but the increase was only statistically significant versus the control group for the navoximod nanoformulation/aPD‐1 combined therapy group (Figure 6g). Although we did not observe statistically significant differences in the population of cytotoxic T‐cells between the single and combination therapy groups, we cannot exclude the impact of immune responses on the observed anti‐tumor effects as reported in previous studies with navoximod. For example, Jiang et al. observed higher but not significant levels of CD4 and CD8 tumor infiltrating T‐cells upon treatment with navoximod.^48^ However, navoximod significantly elevated the levels of INF‐γ and IL‐2 secreted from tumor‐infiltrating lymphocytes.^48^ Since the anti‐cancer immune responses are mediated by different immune system components, our data warrants further research to more comprehensively investigate the impact of the navoximod nanoformulation/aPD‐1 combined therapy on other immune cells and cytotoxic cytokines.

In this study, we did not directly compare the navoximod nanoformulation with the oral formulation of navoximod. Because we anticipate that a substantially higher oral dose would be required to match the anti‐cancer effects yielded by the parenteral navoximod nanoformulation. In the study by Meng et al., oral navoximod was used at 50, 100, and 200 mg, two times per day for 16 days and it was at doses above 100 mg that significant inhibition of kynurenine and therefore anti‐tumor effects were achieved.^49^ Likewise, in the study by Jiang et al., oral navoximod was administered at 0.8 mmol/kg (equivalent to 225 mg/kg) two times per day for the duration of the study.^48^ Therefore, we believe a separate animal study is required only to establish the treatment regimen of oral navoximod to be comparable with the treatment regimen of parenteral navoximod nanoformulation.

## 4 CONCLUSION

The inhibition of immune checkpoints with monoclonal antibodies targeting the PD‐1/PDL1 pathway has significantly impacted the therapeutic landscape, however at present, it only benefits a fraction of patients. There is a critical need for broad spectrum checkpoint inhibition approaches that improve the recruitment of activated effector T‐cells in immunologically cold tumors and address the resistance issue. Inhibition of the IDO pathway is such a promising approach,^50^, ^51^ but clinical trials have been disappointing which likely derived from the poor tumor bioavailability of orally administrated small molecule inhibitors. To address this issue and gain further insight into the therapeutic benefit of combined PD‐1 checkpoint and IDO inhibition, a navoximod nanoformulation was developed using hydrophobic ion pairing. The pairing of the small molecule IDO inhibitor navoximod with sodium deoxycholate efficiently and remarkably increased the loading of the drug within PEG‐PLGA based nanoformulation synthesized using a 3D printed high throughput nanoprecipitation microfluidic chip. The navoximod nanoformulation efficiently inhibited the IDO enzyme in a HNSCC cell line. It synergized with checkpoint inhibitor PD‐1 monoclonal antibodies, significantly enhancing anti‐tumor effects both in vitro and in vivo in an orthotopic murine model of HNC. These effects could be at least partially explained by increased recruitment of cytotoxic CD8 T‐cells. Our study warrants further investigation to fully ascertain the therapeutic benefit of combining IDO inhibition with checkpoint inhibition, such as a direct comparison of navoximod nanoformulation with an oral formulation of navoximod, as well as to rigorously assess the immune related adverse toxicity. The study also confirms that hydrophobic ion pairing is a powerful and highly translatable formulation approach for small molecule drugs with challenging physicochemical properties.

## AUTHOR CONTRIBUTIONS

**Parisa Badiee:** Conceptualization (equal); formal analysis (lead); investigation (lead); methodology (lead); writing – original draft (lead). **Michelle F Maritz:** Methodology (supporting); supervision (supporting); validation (equal); writing – review and editing (equal). **Pouya Dehghankelishadi:** Investigation (equal); methodology (supporting); writing – review and editing (supporting). **Nicole Dmochowska:** Formal analysis (supporting); methodology (supporting); writing – review and editing (supporting). **Benjamin Thierry:** Conceptualization (equal); funding acquisition (lead); supervision (lead); writing – review and editing (equal).

## CONFLICT OF INTEREST STATEMENT

The authors have no conflicts of interest to declare.

### PEER REVIEW

The peer review history for this article is available at https://www.webofscience.com/api/gateway/wos/peer‐review/10.1002/btm2.10599.

## Supporting information


**DATA S1:** Supporting Information.

## Data Availability

The data that support the findings of this study are available from the corresponding author upon reasonable request.
