# Supplementary material for: Hydrophobic ion pairing and microfluidic nanoprecipitation enable efficient nanoformulation of a small molecule indolamine 2, 3‐dioxygenase inhibitor immunotherapeutic
Source: Bioeng Transl Med. 2023 Oct 28;9(1):e10599. doi: 10.1002/btm2.10599 (PMC10771570; doi:10.1002/btm2.10599)
Supplement: Supplementary file 1 — DATA S1: Supporting Information. [file BTM2-9-e10599-s001.pdf]

Supporting information - Hydrophobic Ion Pairing and Microfluidic Nanoprecipitation Enables Efficient Nanoformulation of a Small Molecule Indolamine 2, 3-Dioxygenase Inhibitor Immunotherapeutic

Parisa Badiee [a,b], Michelle F Maritz [a], Pouya Dehghankelishadi [a,b], Nicole Dmochowska [a], Benjamin Thierry [*a]

[a] Future Industries Institute and ARC Centre of Excellence Convergent Bio-Nano Science and Technology, University of South Australia, Mawson Lakes Campus, Adelaide, SA, 5095, Australia

[b] UniSA Clinical and Health Sciences, University of South Australia, City West Campus, Adelaide, SA, 5000, Australia

Supplementary figures

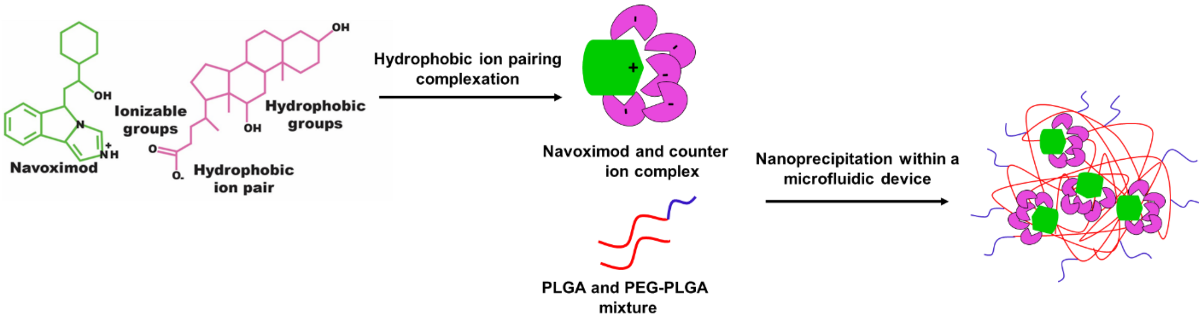

Supplementary figure 1. Schematic illustration of navoximod and sodium deoxycholate hydrophobic ion pairing and encapsulation of the complex within PEG-PLGA nanoparticles by nanoprecipitation.

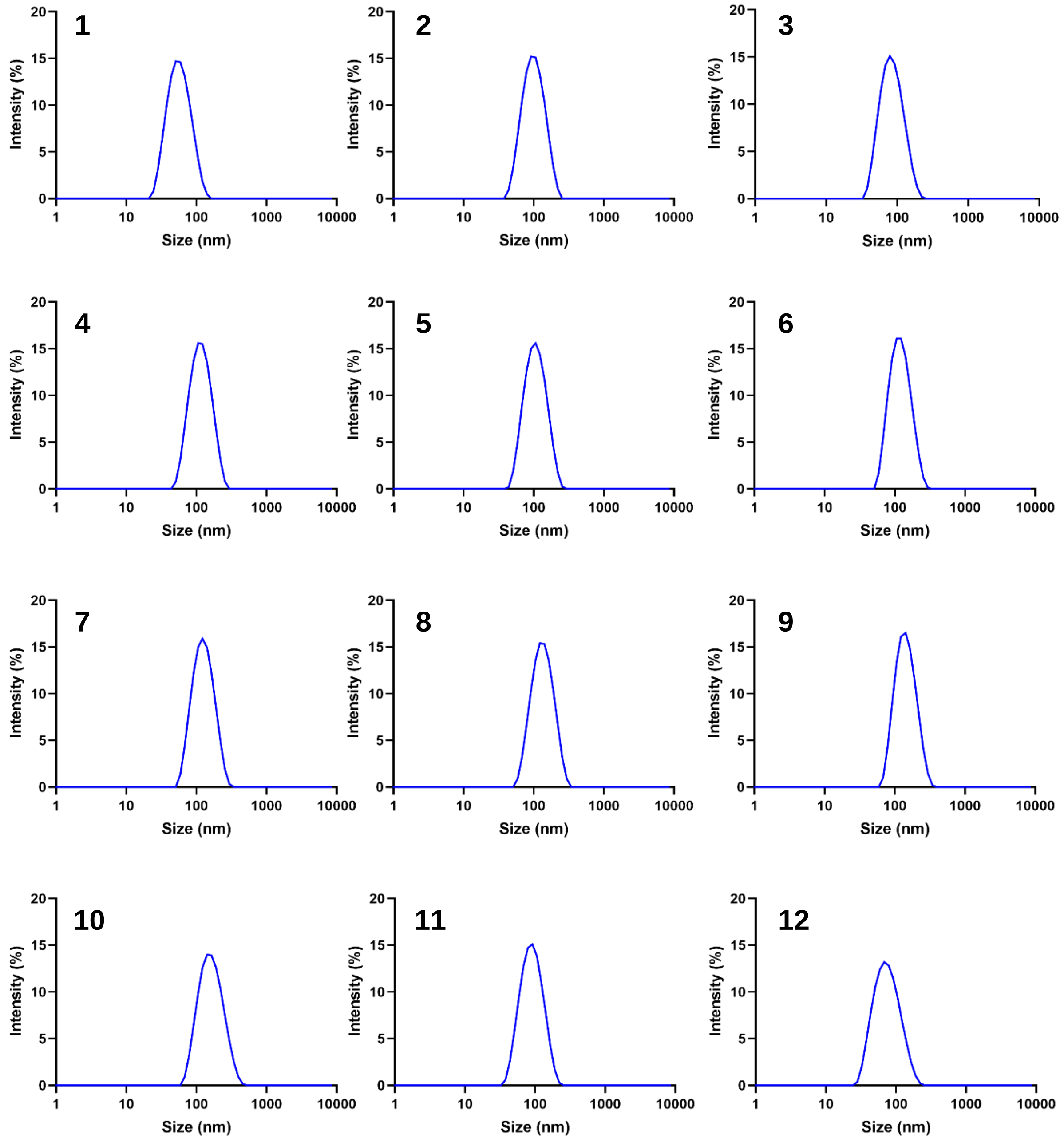

Supplementary figure 2. Hydrodynamic size distribution of navoximod nanoformulations measured in Milli-Q® water using a Zetasizer 3000 HS. 1: navoximod/polymer 20 % (W/W), sodium deoxycholate/navoximod 0 (mol/mol), Aqueous/Solvent flow rate 3/1 (mL/min). 2: navoximod/polymer 10 % (W/W), sodium deoxycholate/navoximod 3/1 (mol/mol), Aqueous/Solvent flow rate 3/1 (mL/min). 3: navoximod/polymer 10 % (W/W), sodium deoxycholate/navoximod 5/1 (mol/mol), Aqueous/Solvent flow rate 3/1 (mL/min). 4: navoximod/polymer 10 % (W/W), sodium deoxycholate/navoximod 10/1 (mol/mol), Aqueous/Solvent flow rate 3/1 (mL/min). 5: navoximod/polymer 20 % (W/W), sodium deoxycholate/navoximod 2/1 (mol/mol), Aqueous/Solvent flow rate 3/1 (mL/min). 6: navoximod/polymer 20 % (W/W), sodium deoxycholate/navoximod 3/1 (mol/mol), Aqueous/Solvent flow rate 3/1 (mL/min). 7: navoximod/polymer 20 % (W/W), sodium deoxycholate/navoximod 4/1 (mol/mol), Aqueous/Solvent flow rate 3/1 (mL/min). 8:

navoximod/polymer 20 % (W/W), sodium deoxycholate/navoximod 5/1 (mol/mol), Aqueous/Solvent flow rate 3/1 (mL/min). 9: navoximod/polymer 20 % (W/W), sodium deoxycholate/navoximod 7/1 (mol/mol), Aqueous/Solvent flow rate 3/1 (mL/min). 10: navoximod/polymer 20 % (W/W), sodium deoxycholate/navoximod 10/1 (mol/mol), Aqueous/Solvent flow rate 3/1 (mL/min). 11: navoximod/polymer 20 % (W/W), sodium deoxycholate/navoximod 5/1 (mol/mol), Aqueous/Solvent flow rate 8/1 (mL/min). 12: navoximod/polymer 20 % (W/W), sodium deoxycholate/navoximod 5/1 (mol/mol), Aqueous/Solvent flow rate 15/3 (mL/min).

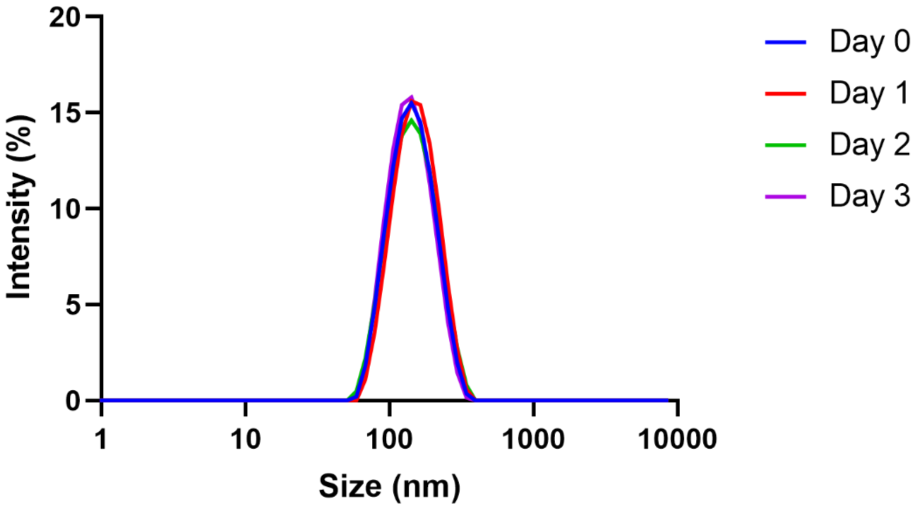

Supplementary figure 3. Hydrodynamic size distribution of the optimised navoximod nanoformulation measured in Milli-Q® water over three days using a Zetasizer 3000 HS.

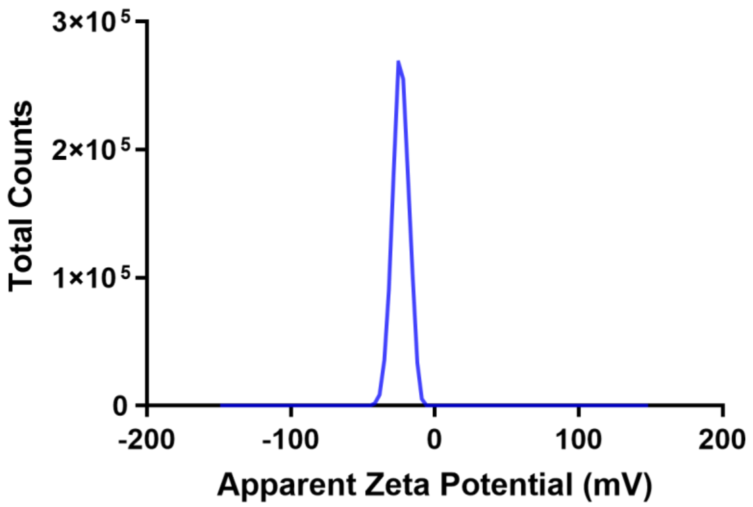

Supplementary figure 4. Zeta potential of the optimised navoximod nanoformulation measured in Milli-Q® water using a Zetasizer 3000 HS.

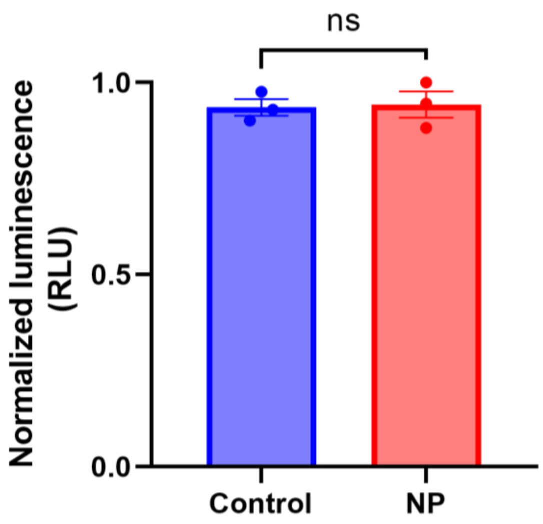

Supplementary figure 5. Effect of PEG-PLGA nanoparticles on the viability of T-cells. Luminescence of metabolically active T-cells two days after treatment by PEG-PLGA nanoparticles (NP) or PBS (control), using RealTime-Glo™ MT cell viability assay and measured at 490 nm by plate reader. ns indicates not significant by t-test.

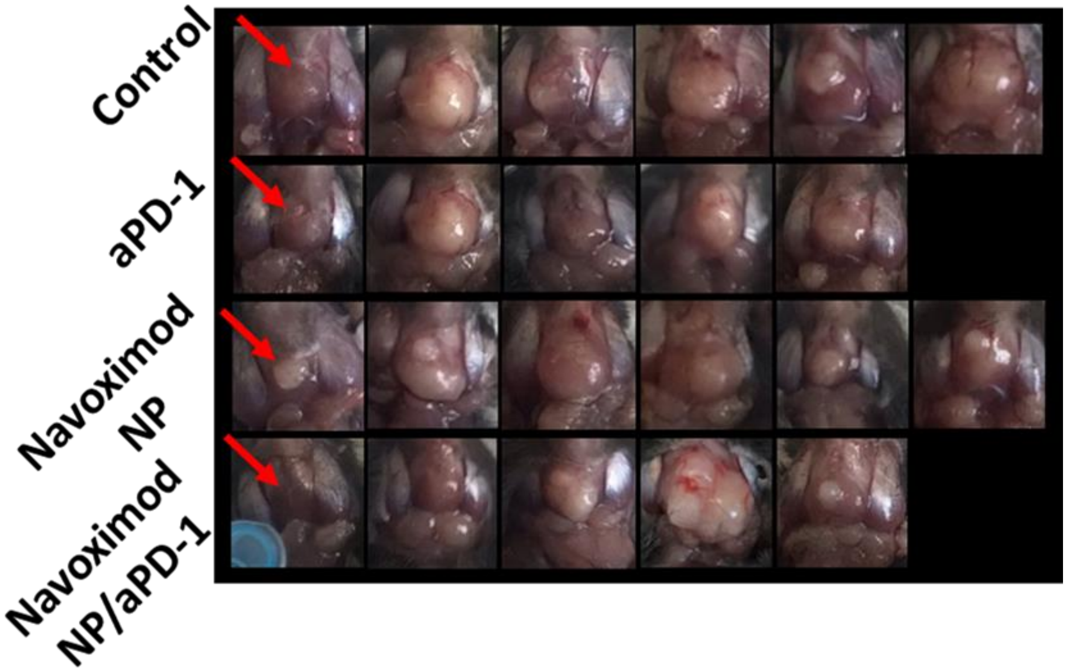

Supplementary figure 6. Images of tumors at the endpoint after incision of the skin at the cervical region. The red arrows show the location of the tumors.

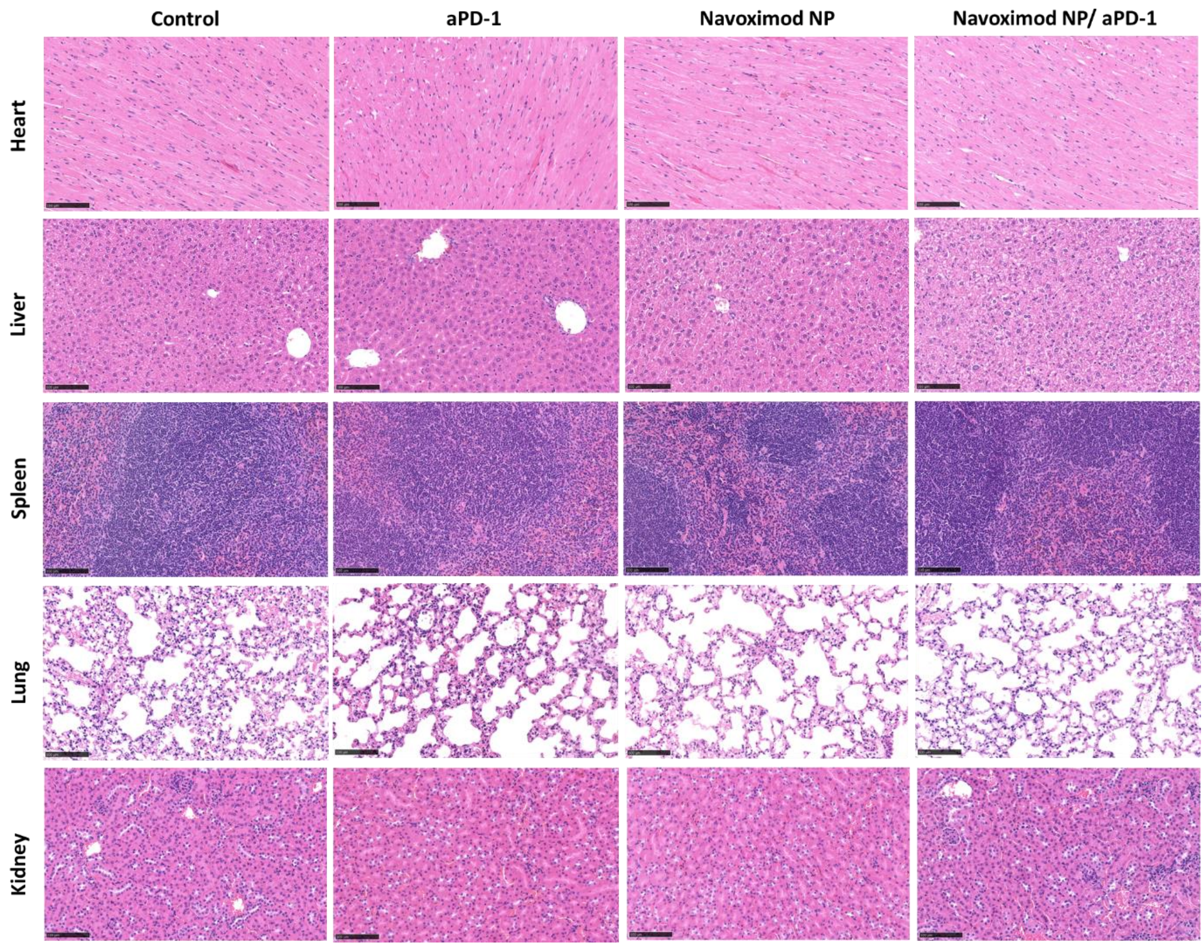

Supplementary figure 7. Histological hematoxylin and eosin staining of heart, liver, spleen, lung and kidney of tumor bearing mice following two weeks of treatment with saline (control), aPD-1, navoximod nanoformulation and combined navoximod nanoformulation/ aPD-1. The scale bar is 100 µm.

Methods

Physicochemical characterization of navoximod nanoformulation

The hydrodynamic size of the navoximod nanoformulation was determined using dynamic light scattering (in Milli-Q® water) using a Zetasizer 3000 HS (Malvern Instruments Ltd, UK). The hydrodynamic size measurement was also repeated over three days for the optimised navoximod nanoformulation. The zeta potential of the nanoparticles (NPs) was measured with the Zetasizer 3000 HS (in Milli-Q® water). The morphology of the navoximod nanoformulation was assessed after negative staining with 2 % phosphotungstic acid, using a transmission electron microscope (TEM) (JEM-2100F-HR, Jeol, Japan).

Reversed phase high-performance liquid chromatography (HPLC) was used to evaluate the navoximod loading using an Agilent 1200 HPLC system and Eclipse Plus C18 column (4.6 x 100 mm, 3.5 µm) (Agilent Technologies, USA). After centrifugation, pelleted navoximod nanoformulation was dissolved in acetonitrile. A gradient mobile phase of water and HPLC grade methanol with formic acid (0.1 %, Sigma, USA) was used. The mobile phase was pumped at a flow rate of 2 mL/minute with 10 % methanol/ 90 % water, which was switched to 90 % methanol/10 % water over 8 minutes. Subsequently, the drug peak was detected by DAD UV-Vis spectroscopy at 262 nm. A standard curve of navoximod in acetonitrile (0.5 µg/mL to 100 µg/mL) was prepared. The percentage of drug loading was calculated using the equation below (equation 1).

$$\text{Drug loading (\%)} = \frac{\text{Mass of loaded navoximod}}{\text{Mass of navoximod nanoformulation}} \times 100.$$

(Equation 1)

The mass of the navoximod nanoformulation was obtained following freeze drying (Modulyo® freeze dryer, ThermoFisher, USA).

The release profile of the navoximod nanoformulation was measured using dialysis. 0.3 mL of navoximod nanoformulation (equal to 0.6 mg/mL of navoximod) suspension in 1x Dulbecco's PBS (DPBS) (ThermoFisher, USA) was transferred into a Membra-cel® MD34 14X100 CLR dialysis tube (Sigma Aldrich, USA). The dialysis bag was placed into a beaker of 20 mL DPBS as the releasing medium, while stirring at 100 rpm. The release study was performed at both room temperature and 37°C to simulate storage at shelf temperature and in vivo temperature, respectively. The volume of the sample and releasing medium were adjusted based on the

solubility of navoximod to mimic the sink condition and prevent the saturation of dissolution. At 0.5, 1, 2, 4, 6, 8 and 24 h time-points, 1 mL of the releasing media was collected to quantify the released drug, after which, the medium was replaced with 20 mL of fresh DPBS to maintain the sink condition. The sample was then centrifuged (24000 g, 15 minutes, 4 °C) and the resulting supernatant was analysed using HPLC. The percentage of released navoximod was calculated based on the total amount of navoximod in the nanoformulation at the start of the release study, which was measured by dissolving the nanoparticles in acetonitrile and quantifying navoximod by HPLC. The percentage of the cumulative release of navoximod was reported.

Cell culture

UM-SCC1 cells (Merck Milipore, USA) were utilised for in vitro testing and co-cultured with T-cells for cytotoxicity assays.
For cell cycle analysis, fucci-SCC1 was prepared as previously reported (fucci construct from RIKEN BRC, Japan) [1]. Fucci cells fluoresce green and red in S-G2 and G1 phases, respectively. UM-SCC1 cells were cultured in complete RPMI media, supplemented with 10 % fetal bovine serum, 1 % streptomycin/penicillin and 1 % L-glutamine (all Gibco, UK).

The murine oral carcinoma-1 (MOC-1) (Kerafast Cell Bank Repository, USA) cell line was used which originates from a murine primary tumor of the oral cavity [2]. MOC-1 cells were cultured in 2:1 IMDM to Ham's F12 nutrient mixture (ThermoFisher, USA), supplemented with 5% fetal bovine serum (GE Healthcare, USA), 5 µg/L epidermal growth factor (EMD Millipore, USA), 5 mg/L insulin, 40 µg/L hydrocortisone (both Sigma Aldrich) and 1% streptomycin/penicillin. Cells were grown in 90% humidity and 5% CO_2 at 37˚C and were split when at 80% confluency using 0.25% trypsin-EDTA.

IDO enzymatic activity

To analyse the efficacy of the navoximod nanoformulation to decrease IDO activity, UM-SCC1 cells were seeded in a 96-well plate ($5x10^3$ cells per well). After an overnight incubation, the cell culture media was replaced with complete RPMI supplemented with INF-γ (400 ng/mL) to stimulate IDO expression. The cells were treated with several concentrations of navoximod nanoformulation or free navoximod from 0.001 µM to 20 µM. After 72 h of treatment, 150 µL

of supernatant was collected from each well and incubated with 75 µL trichloroacetic acid (30 %) at 50 °C for 30 minutes to hydrolyse N-formylkynurenine to kynurenine. The samples were then centrifuged for 5 minutes at 5000 g and 100 µL of the supernatant was then combined with an equal volume of Ehrlich's reagent (2% *p*-dimethylamino-benzaldehyde w/v in glacial acetic acid) to develop yellow colour. Various concentrations of kynurenine (0 - 240 µM) were used to generate a standard curve for the quantification of kynurenine in the samples. The absorbance of the sample was then assessed at 490 nm using a plate reader (FLUOstar OPTIMA, BMG Labtech, USA).

Impact of navoximod nanoformulation on cancer cells metabolic activity

The effect of navoximod nanoformulation or blank NPs on the metabolic activity of UM-SCC1 cells was assessed based on the conversion of MTT salt (Sigma Aldrich, USA) to formazan. UM-SCC1 cells were seeded in a 96-well plate ($7.5x10^3$ cells per well). After an overnight incubation, the cell culture media was changed and the cells were treated with various concentrations of navoximod nanoformulation or equal doses of blank NPs (navoximod doses ranging from 0 – 100 µM). After 48 h, the cell culture media was discarded, and the cells were incubated in 60 µL complete RPMI and 40 µL MTT solution (5 mg/mL). After 4 h, the MTT solution was replaced with 100 µL DMSO to solubilize formazan as the product of MTT reduction. The developed colour was measured at 540 nm using a plate reader (FLUOstar OPTIMA, BMG Labtech, USA).

T-cell isolation and activation

Following written consent, peripheral blood was collected from healthy volunteers (University of South Australia Human Research Ethics Committee approval 201726).

Primary T-cells were enriched by RosetteSep™ human T-cell enrichment kit (Stemcell) following the manufacturer's instructions. The isolated T-cells (10^6 cells/mL) were activated for 2 days using an anti-CD3 (5 µg/mL) coated well plate with anti-CD28 (4 µg/mL) and IL-2 (500 U/mL).

T-cell cytotoxicity modulation by navoximod nanoformulation /aPD-1

UM-SCC1 cells (10^5) were seeded in a 24-well plate and incubated in complete RPMI media supplemented with INF-γ (400 ng/mL). After an overnight incubation, the cell culture medium was changed to complete RPMI supplemented with IL-2 (10 U/mL) and INF-γ (50 ng/mL) and the cancer cells were co-cultured with 2×10^5 activated T-cells. The cells were then treated with navoximod nanoformulation (10 µM) or aPD-1 (5 µg/mL) or a combination of navoximod nanoformulation / aPD-1 or no treatment which was used as the control.

Imaging flow cytometry was used to determine the expression of various T-cell markers. Co-cultured T-cells were washed twice with DPBS and centrifuged at 500 g for 5 minutes. The cells were stained for 30 minutes with specific fluorophore conjugated antibodies in bovine serum albumin (0.5%) in DPBS. After staining, the cells were washed twice with DPBS and analysed by imaging flow cytometry Image Stream X (AMNIS, USA). Imaging flow cytometry data was analysed using IDEAS 6.2 (EMD Millipore, USA) and FlowJo V10 (FlowJo, USA). The following human antibodies were used for immune-staining: FITC CD3 (ThermoFisher, USA), BV421 CD3, PE PD-1, FITC CD4, PerCP Cy5.5 CD8, AF647 Ki67, AF647 foxP3 (BioLegend, USA) and BV421 granzyme B (BD Bioscience, USA). Ki67, foxP3 and granzyme B were detected after intracellular staining using Foxp3 / Transcription Factor Staining Buffer kit (ThermoFisher, USA), as per the manufacturer's recommendations.

The population of immunosuppressive Treg, was analysed by imaging flow cytometry 48 h after treatment of co-cultures and following immunostaining for CD3, CD4 and foxP3.

The effect of treatment on T-cell proliferation was evaluated by CellTrace™ CFSE Cell Proliferation Kit (Thermo Fisher Scientific, USA). Activated T-cells were labelled with CellTrace™ CFSE Cell Proliferation Kit according to the manufacturer's conditions and co-cultured with UM-SCC1 cells. Approximately 48 hours after treatment, the T-cells were collected and the CFSE intensity was quantified by imaging flow cytometry after gating for $CD3^+$ single cells. In this method, CFSE dilution indicates T-cell proliferation. T-cell proliferation was further investigated by examining Ki67 expression. One day after treatment, fluorescent intensity of Ki67 on T-cells was quantified by imaging flow cytometry after staining with Ki67 and CD3 antibodies.

The efficacy of navoximod nanoformulation or navoximod nanoformulation /aPD-1 on inhibiting PD-1 receptors as a marker of T-cell exhaustion and granzyme B as a marker of T-cell cytotoxicity was determined by quantifying PD-1's fluorescence of $CD3^+$ cells 48 h after

treatment and granzyme B's fluorescence of $CD3^+$ T-cells, 24 h after treatment by imaging flow cytometry.

To exclude the impact of the nanoparticles on T-cell's viability and metabolic activity, the metabolic activity of the activated T-cells was determined by RealTime-Glo™ MT cell viability assay (Promega, US) following treatment with drug-free PEG-PLGA nanoparticles or PBS as a control for two days. The luminescence of metabolically active T-cells was quantified using a FLUOstar OPTIMA plate reader (BMG Labtech, USA) equipped with a 590 nm emission filter.

Cancer cell apoptosis and cell cycle function by navoximod nanoformulation /aPD-1

UM-SCC1 cells were seeded in an 8 well slide (Ibidi GmbH, Germany), 2×10^4 cells/ well and co-cultured with 4×10^4/ well activated T-cells. The cells were treated for 24 h with navoximod nanoformulation (10 µM) or aPD-1 (5 µg/mL) or a combination of navoximod nanoformulation / aPD-1 or no treatment, as the control. The same dose of free navoximod was also used as the positive control. To determine the rate of cancer cells apoptosis, CellEvent™ Caspase-3/7 Green Detection Reagent (ThermoFisher, USA) was used following the manufacturer's instructions. This reagent is a DNA binding fluorescent dye conjugated to a DEVD peptide. The peptide is cleaved by activated caspase 3/7, allowing the dye to bind to DNA and show fluorescence. The cells were imaged with a Confocal Laser Scanning Microscope 710 (Zeiss, Germany) using objective 10x and the FITC channel. The number of apoptotic cells was enumerated using NIS-Elements software (Nikon, Japan).

Fucci-SCC1 cells were used in the co-culture system to investigate the effects of T-cells on the cancer cell cycle after treatment with navoximod nanoformulation or navoximod nanoformulation / aPD-1 as described above. The cells were imaged by Confocal Laser Scanning Microscope 710 (Zeiss, Germany) using objective 10x and in the FITC and Texas red channels. The number of red and green cells were counted using NIS-Elements software (Nikon, Japan), as the indicator of G1 and S-G2 cell cycle, respectively, and the green/red cells ratio was calculated.

Preclinical investigation of combined checkpoint inhibition and treatment with the navoximod nanoformulation

Briefly, for the orthotopic model, 300,000 MOC-1 cells were resuspended in 1X DPBS and mixed with Cultrex Basement Membrane Extract Type 3 (R&D systems, USA), in a 1:1 mixture. Using an insulin syringe (BD, USA), these cells were injected into the floor of the mouth of six-week old C57BL/6J mice of either sex. Tumor growth was observed by measuring tumor volume using digital callipers to measure two perpendicular diameters. The volume was calculated as volume = 0.5 × length × width^2. A maximum tumor cut off size of 256 mm^3 or 8 mm in one diameter was set. When the tumors reached approximately 50 mm^3, animals were divided into four groups (N=5 or 6/group) to receive the following treatment regimen: Control (saline, five times per week, 10 injections), aPD-1 (10 mg/kg, two times per week, 4 injections), navoximod nanoformulation (10 mg/kg, three times per week, 6 injections) and combined navoximod nanoformulation/aPD-1 (10 mg/kg for both treatment, five times per week, 10 injections). The navoximod nanoformulation was injected by the intravenous (IV) route through the lateral tail vein, aPD-1 was injected by the intraperitoneal (IP) route and saline was injected both IV and IP as a control. The dosing regimen was chosen to be in line with the previous studies [3,4]. For this animal study, a large batch of the navoximod nanoformulation was prepared using the protocol explained earlier and stored at -80 °C in the presence of trehalose (equivalent to the mass of polymer) for later use.

The anti-tumor efficacy of the treatment was investigated by measuring the tumor volume three times per week. The tumor growth inhibition was determined using equation 2 [5], where Tt and T0 are the geometrical means of tumor volume of a treated group at time t and the start of treatment, respectively. Ct and C0 are the same values in the control group.

$$Tumour\ growth\ inhibition = \frac{1-(Tt/T0)/(Ct/C0)}{1-(C0/Ct)} \times 100$$

(Equation 2)

The combination index of the treatment was calculated using the Chou and Talalay formula [6], (equation 3), where a combination index < 1 indicates a synergistic effect.

$$Combination\ index = \frac{[(Navoximod\ nanoformulation\ effect)^{-1} + (aPD-1\ effect)^{-1}]-1}{(Navoximod\ nanoformualtion\ and\ aPD-1\ combination\ effect)^{-1}}$$

(Equation 3)

Three days after the last treatment, the animals were humanely culled and the tumors were excised and weighed. The harvested tumor tissues were mechanically minced and dissociated into single cells by incubating in a dissociation cocktail for 45 minutes at 37°C and shaking at 400 rpm. The dissociation cocktail consisted of collagenase (300 U/mL), hyaluronidase (100 U/mL, Stem-Cell Technologies, USA), DNase I (0.1 mg/mL, Sigma Aldrich, USA) and

TrypLE^{TM} express enzyme (0.1X, Thermofisher, USA). The cell suspension was filtered by a 70 µm cell strainer and washed. The resulting single cell suspensions were stained with the viability stain 575V (BD Biosciences, USA) for 15 minutes and then Fc blocked with CD16/CD32 antibody (BD Biosciences) for 5 minutes followed by staining with BV786 CD3 and APC-H7 CD8 in Brilliant Stain Buffer (BSB, BD Biosciences). Samples were analyzed using a Fortessa flow cytometer (BD Biosciences) and analyzed with FlowJo (FlowJo, USA). Major organs including the heart, liver, spleen, lung and kidney were collected, fixed by 4 % paraformaldehyde and stained with hematoxylin and eosin for toxicity assessment of the treatment and a veterinary pathologist (Gribbles Veterinary Pathology, Adelaide, Australia) was consulted.

References

[1] A. Sakaue-Sawano, T. Kobayashi, K. Ohtawa, A. Miyawaki, Drug-induced cell cycle modulation leading to cell-cycle arrest, nuclear mis-segregation, or endoreplication, BMC Cell Biology. 12 (2011) 1–12. https://doi.org/10.1186/1471-2121-12-2.

[2] N.P. Judd, A.E. Winkler, O. Murillo-Sauca, J.J. Brotman, J.H. Law, J.S. Lewis, G.P. Dunn, J.D. Bui, J.B. Sunwoo, R. Uppaluri, ERK1/2 regulation of CD44 modulates oral cancer aggressiveness, Cancer Research. 72 (2012) 365–374.

[3] K. Luo, Y. Lian, M. Zhang, H. Yu, G. Wang, J. Li, Charge convertible biomimetic micellar nanoparticles for enhanced melanoma-targeted therapy through tumor cells and tumor-associated macrophages dual chemotherapy with IDO immunotherapy, Chemical Engineering Journal. 412 (2021). https://doi.org/10.1016/j.cej.2021.128659.

[4] B. Feng, F. Zhou, B. Hou, D. Wang, T. Wang, Y. Fu, Y. Ma, H. Yu, Y. Li, Binary Cooperative Prodrug Nanoparticles Improve Immunotherapy by Synergistically Modulating Immune Tumor Microenvironment, Advanced Materials. 30 (2018) 1803001. https://doi.org/10.1002/adma.201803001.

[5] F.I. Huang, Y.W. Wu, T.Y. Sung, J.P. Liou, M.H. Lin, S.L. Pan, C.R. Yang, MPT0G413, A novel HDAC6-selective inhibitor, and bortezomib synergistically exert anti-tumor activity in multiple myeloma cells, Frontiers in Oncology. (2019). https://doi.org/10.3389/fonc.2019.00249.

[6] T.C. Chou, P. Talalay, A simple generalized equation for the analysis of multiple inhibitions of Michaelis-Menten kinetic systems, Journal of Biological Chemistry. (1977). https://doi.org/10.1016/s0021-9258(17)39978-7.
